# Three-year follow-up of high-risk keratoplasty following fine-needle diathermy of corneal neovascularization combined with bevacizumab

**DOI:** 10.1007/s00417-021-05546-w

**Published:** 2022-01-06

**Authors:** Mert Mestanoglu, Alexander Händel, Claus Cursiefen, Deniz Hos

**Affiliations:** 1 Department of Ophthalmology, Faculty of Medicine and University Hospital Cologne, University of Cologne, Cologne, Germany; 2 Center for Molecular Medicine Cologne (CMMC), University of Cologne, Cologne, Germany


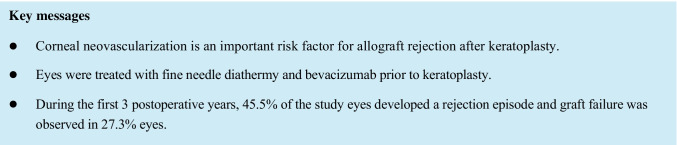


Dear Editor,

Pathologically vascularized corneas are at high risk for immune reactions (IR) and subsequent graft failure after penetrating keratoplasty (PK) [1]. Thus, the inhibition of corneal neovascularization seems to be a feasible strategy to improve graft survival after high-risk PK. Several approaches such as fine-needle diathermy (FND), pharmacological interference, e.g., with the vascular endothelial growth factor (VEGF) pathway, and others have been used to reduce IR episodes and maintain rejection-free graft survival [2].

Previously, our group reported that FND combined with subconjunctival bevacizumab treatment prior to PK results in rejection-free graft survival rates of 92.9% after 1 year and 78.4% after 3 years. The mean follow-up time in that study was 560 days [3]. However, long-term results of this approach are so far unknown.

Here, we report 3-year follow-up results of a cohort of 22 vascularized eyes treated with FND in combination with subconjunctival bevacizumab before high-risk PK with a mean follow-up of 1483 days. All patients were followed up for at least 36 months, and IR episodes and graft failures were retrospectively evaluated. An IR was defined by the presence of at least one of the following criteria: new endothelial precipitates or Khodadoust line on the graft, new anterior chamber cells/flare, or new focal/diffuse edema of the graft. Distribution of time-to-event data was described by the Kaplan–Meier method using SPSS Statistics 27 (IBM Corp., Armonk, NY, USA).

**Fig. 1:**
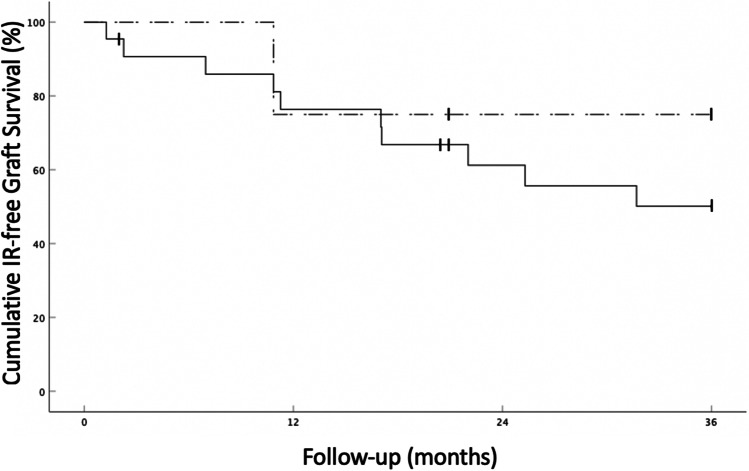
Continuous line: Kaplan–Meier curve depicting IR-free corneal allograft survival rates in high-risk penetrating keratoplasty patients treated with pretransplant fine-needle diathermy combined with subconjunctival bevacizumab (all study eyes, *n* = 22). Vertical dashes indicate censored observations. Dashed line: Kaplan–Meier curve depicting IR-free corneal allograft survival rates in eyes where complete regression of corneal neovascularization was achieved prior to keratoplasty (*n* = 4)

**Fig. 2:**
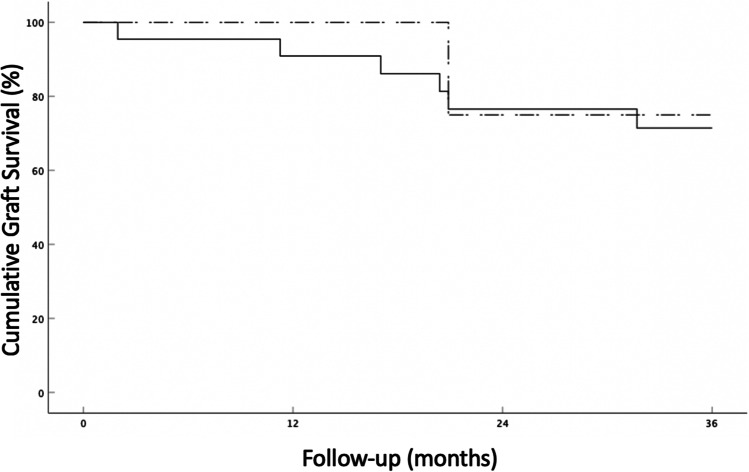
Continuous line: Kaplan–Meier curve depicting corneal allograft survival rates in high-risk penetrating keratoplasty patients treated with pretransplant fine-needle diathermy combined with subconjunctival bevacizumab (all study eyes, *n* = 22). Dashed line: Kaplan–Meier curve depicting corneal allograft survival rates in eyes where complete regression of corneal neovascularization was achieved prior to keratoplasty (*n* = 4)

Our cohort consisted of 11 male and 11 female patients; the mean patient age was 57.1 years. Three patients had preexisting corneal neovascularization in one quadrant, two patients in two quadrants, and 17 patients in three or four quadrants before treatment. Ten patients (45.5%) had a history of a previous graft. Eighteen eyes (81.8%) required additional FND and bevacizumab on the day of PK, whereas four eyes (18.2%) showed complete vessel regression with no need of repeat FND and bevacizumab. The mean time between (last) FND and bevacizumab treatment and high-risk PK was 114.5 days (range 0–794 days). During follow-up, an IR episode was recorded in ten eyes (45.5%) (Fig. 1). In six eyes (27.3%), graft failure was observed (Fig. 2). Three of these eyes with graft failure had a previous IR episode, whereas in the other three eyes, an IR episode did not precede graft failure. Five of these eyes had a history of a previous corneal graft (83.3%). In eyes where complete vessel regression was observed before PK (*n* = 4), only 1 IR episode (25%) (Fig. 1) and 1 graft failure with no previous IR episode (25%) (Fig. 2) were observed.

Our study demonstrates a relatively high IR rate (45.5%), which may be due to the presence of residual vessels at the day of PK in most of the patients. The 3-year graft survival rate of 72.7% is similar to the rate of the Australian Graft Registry 2015 report, which had demonstrated a survival rate of 70% for corneas with corneal neovascularization [4].

In the subgroup of our cohort that did not require simultaneous FND and bevacizumab at time of transplantation because of complete vessel regression, only one IR episode was observed, resulting in a rejection-free 3-year graft survival rate of 75%. This rate seems to be considerably higher than the IR-free survival rate of the eyes requiring simultaneous FND and bevacizumab treatment at the day of PK. Thus, our results implicate that complete vessel regression prior to keratoplasty should be aimed for, although a definitive conclusion cannot be drawn due to the small group size.

Further limitations of our study include its retrospective nature, heterogeneity of the time between (last) FND/bevacizumab treatment and PK, and the lack of an internal control group. Nevertheless, to our knowledge, this is the longest follow-up reported in eyes treated with FND and bevacizumab prior to high-risk PK.

An attempt to achieve complete vessel regression appears as a viable strategy for rejection-free graft survival. Further research on more potent angioregressive methods such as corneal crosslinking is required to better define the contribution of complete vessel regression to rejection-free corneal allograft survival [5].
